# Establishment of a Swine Model for Validation of Perfusion Measurement by Dynamic Contrast-Enhanced Magnetic Resonance Imaging

**DOI:** 10.1155/2014/390506

**Published:** 2014-02-27

**Authors:** Anika Sauerbrey, Stefan Hindel, Marc Maaß, Christine Krüger, Andreas Wissmann, Martin Kramer, Benno Nafz, Lutz Lüdemann

**Affiliations:** ^1^Radiation and Tumor Clinic, Essen University Hospital, Hufelandstraße. 55, 45147 Essen, Germany; ^2^Evangelisches Krankenhaus Wesel GmbH, Schermbecker Landstraße 88, 46485 Wesel, Germany; ^3^Central Animal Laboratory, Essen University Hospital, Hufelandstraße. 55, 45147 Essen, Germany; ^4^Department of Small Animal Surgery, Hospital of Veterinary Medicine, Justus Liebig University Giessen, Frankfurter Straße 94, 35392 Gießen, Germany; ^5^Charité Universiätsmedizin Berlin, Charitéplatz 1, 10117 Berlin, Germany

## Abstract

The aim of the study was to develop a suitable animal model for validating dynamic contrast-enhanced magnetic resonance imaging perfusion measurements. A total of 8 pigs were investigated by DCE-MRI. Perfusion was determined on the hind leg musculature. An ultrasound flow probe placed around the femoral artery provided flow measurements independent of MRI and served as the standard of reference. Images were acquired on a 1.5 T MRI scanner using a 3D T1-weighted gradient-echo sequence. An arterial catheter for local injection was implanted in the femoral artery. Continuous injection of adenosine for vasodilation resulted in steady blood flow levels up to four times the baseline level. In this way, three different stable perfusion levels were induced and measured. A central venous catheter was used for injection of two different types of contrast media. A low-molecular weight contrast medium and a blood pool contrast medium were used. A total of 6 perfusion measurements were performed with a time interval of about 20–25 min without significant differences in the arterial input functions. In conclusion the accuracy of DCE-MRI-based perfusion measurement can be validated by comparison of the integrated perfusion signal of the hind leg musculature with the blood flow values measured with the ultrasound flow probe around the femoral artery.

## 1. Introduction

Perfusion is the supply of tissues with blood. Specific perfusion is defined as the volume of blood flowing through an organ or tissue per unit of time and is usually given as mL/(g∗min⁡) or mL/(cm^3^∗min⁡). Specific perfusion is an important parameter for characterizing tumors and their microenvironments. In oncological patients, increased perfusion often indicates neoangiogenesis within a tumor. The amount of tumor perfusion has been shown to correlate with the degree of malignancy [1]. Additionally, perfusion also determines the radiosensitivity of malignant tumors. A high oxygen level supplied by high perfusion may double or triple the destructive effects of ionizing radiation. This so-called oxygen effect is based on free oxygen molecules, which release reactive radicals that cause cell damage. Consequently, hypoxia reduces radiation sensitivity. Studies in patients with head and neck cancer and cervical cancer show that increased hypoxia in the tumor reduces local tumor control and survival. The oxygen supply to a tumor depends on several factors, including vascular abnormalities, intratumoral pressure gradient, and acute or chronic anemia [2]. Hypoxic areas are mainly seen in large tumors with internal necrosis. However, small tumors, surgical margins, areas of tumor invasion, and micrometastases may also be hypoxic [2].

There are several methods for measuring perfusion in biological tissues. Most techniques use indicator agents that are added to the blood. The distribution of the indicator provides indirect information on the distribution of the blood. For the measurement of perfusion using positron emission tomography (PET), radioactive markers (e.g., H_2_^15^O, ^18^F-fluorodeoxyglucose, and ^133^Xe) are used. For determination of perfusion with computed tomography (CT), X-ray contrast media are used. Both methods expose the patient to ionizing radiation.

Furthermore, microspheres can be used for perfusion measurements in animal models. Microspheres are colored or radioactively labeled polystyrene beads with diameters of 5–50 *μ*m. After systemic administration, they are dispersed in the bloodstream and become lodged in the capillaries by microembolization. At the same time, a blood sample has to be taken some distance distal to the site of injection. This blood sample serves as a reference for calculating blood flow. Next, tissue samples have to be taken from the organ under investigation. From the ratio of the number of microspheres contained in the tissue sample and the reference blood sample, regional blood flow at the time of measurement can be calculated. This method is very reliable and widely employed [3, 4]. A disadvantage of this method is that perfusion can be determined only after but not during the experiment. Moreover, it is not possible to set a certain blood flow by administering a vasodilator.

Increasingly, perfusion measurement is performed using magnetic resonance imaging (MRI). Several MRI techniques are available for perfusion measurement. The most important methods are dynamic contrast-enhanced MRI (DCE-MRI) and arterial spin labeling (ASL). Arterial spin labeling uses blood as an endogenous marker. Radiofrequency pulses are applied to invert or saturate the longitudinal magnetization of arterial blood upstream of the region of interest. The labeled blood causes a signal reduction in the target tissue. Images with and without labeled blood are acquired to generate subtraction images for determination of perfusion [5].

DCE-MRI uses a contrast medium (CM) that is injected as an intravascular bolus. Perfusion can then be determined from the resulting temporal changes in CM concentrations in blood and tissue. The accuracy of perfusion measurement by DCE-MRI is unclear. In addition to perfusion, vascular permeability as a functional parameter can be determined by measuring CM extravasation. An additional aim is to assess the interstitial and vascular volumes.

As a highly perfused organ, the kidney has been used to evaluate perfusion measurement with DCE-MRI [6]. However, since tumor perfusion is significantly lower than kidney perfusion, the method of DCE-MRI needs to be investigated in an organ/tissue with lower perfusion as well. Pigs are very similar to humans in terms of anatomy and physiology. Therefore, the pig is very suitable as a model for evaluating perfusion measurement using MRI. Vessel sizes and the proportions of muscles are comparable to those of man. For perfusion quantification using DCE-MRI it is important that the animal model has a similar heart rate and that similar volumes of CM are injected. Small animals, such as mice and rats, have much higher circulation rates than humans. Consequently, MRI-based perfusion-measuring techniques used in small animals are only partially transferable to humans. For DCE-MRI perfusion measurement, a high temporal resolution is required. Additionally, a relatively large spatial coverage is needed to cover the volume of a human tumor. An optimal balance between spatial resolution, spatial coverage, and temporal resolution is desired.

Compared to other organs, such as kidney or brain, muscle perfusion is relatively low at rest and varies widely during physical activity. Therefore, muscles are well suited to investigate how well different perfusion levels can be detected and quantified by DCE-MRI. Pigs have a large muscle mass due to selective breeding for meat.

The gold standard used for blood flow measurement in the present study is an ultrasonic transit time flow probe that is fully independent of the perfusion imaging technique. It can be surgically attached to the artery of the supply area to be measured and it enables continuous measurement of pulsatile blood flow through this artery. Unlike electromagnetic or Doppler systems, the flow probe is insensitive to hematocrit and particulate matter. The transit time technique yields absolute flow values, whereas other systems measure only flow changes [7]. The flow probe consists of two ultrasonic transducers, each with a piezocrystal and a metal reflector. The vessel is placed between the transducers and the reflector. The two transducers serve to transmit and receive sound waves. During the upstream cycle, the sound wave travels against the direction of blood flow. Depending on the flow amount, the total transit time is increased. During the downstream cycle, the sound wave travels with the flow of blood, which results in a decrease in total transit time for the same flow amount. Using wide-beam ultrasonic illumination, the Transonic flowmeter subtracts the downstream transit time from the upstream transit time. This difference between the integrated transit times is a measure of volume flow (mL/min) rather than velocity [8]. The accumulated perfusion signal measured by MRI corresponds to the blood flow of the artery of the supply area measured by the ultrasonic flow probe.

To perform measurements beyond baseline flow, blood flow is enhanced by a locally injected vasodilator. The antiarrhythmic drug adenosine is especially well suited for this purpose. Adenosine consists of the nucleotide base adenine and the sugar *β*-D-ribose. Adenosine receptors are present in vascular smooth muscle and endothelium [9]. Adenosine has a very short plasma half-life well below 10 seconds. Vasodilation is observed within 30 seconds of peripheral venous administration. A faster effect is expected after injection into the central circulation. To maintain the effect of adenosine over longer periods, it should be administered continuously, for example, via a syringe pump. The vasodilatory effect of adenosine lowers blood pressure. Unlike many other vasodilators, adenosine administration rarely leads to reflex tachycardia and renin secretion. Adenosine increases atrioventricular conduction time and reduces heart rate. It is used therapeutically in paroxysmal supraventricular tachycardia (e.g., to treat AV nodal reentrant tachycardia) [10].

## 2. Material and Methods

### 2.1. Test Animals

We chose pigs of similar body size as humans in order to establish a well-suited model for clinical applications. A total of 8 female pigs, German landrace or hybrid form (body weight, 56–74 kg), were investigated to implement the method. Only healthy animals without known cardiovascular or musculoskeletal disorders were used. The pigs were not fed overnight prior to the experiment but had free access to water. All experiments were reviewed and approved by the local animal protection committee and performed according to the rules of the German animal welfare regulations.

### 2.2. Surgical Technique: Implantation of Ultrasound Flow Probe, Arterial Catheter, and Central Venous Catheter

All surgical procedures were performed under aseptic conditions in an operating room equipped for large animals.

Premedication was performed using 30 mg/kg ketamine (ketamine 10%, Ceva Tiergesundheit GmbH, Germany), 2 mg/kg azaperone (Stresnil Janssen-Cilag GmbH, Germany), and 0.02–0.05 mg/kg atropine sulfate (Atropinsulfat, B. Braun Melsungen AG, Germany) by intramuscular injection.

A peripheral 20-G venous catheter was placed in an ear vein and, about 30 min after premedication, total intravenous anesthesia was started. A perfusor was used to inject 4–7 mg/kg/h propofol (Propofol-ratiopharm, Ratiopharm, Germany), 0.1–0.5 mg/kg/h midazolam (midazolam injection solution 0.5%, Germany), and 0.0015 mg/kg/h fentanyl (fentanyl citrate solution 3.925 mL/50 mL, Germany). A tracheal tube was placed (Hi-Contour cuffed tracheal tube, ID 8.0, Mallinckrodt, Ireland), and the pig was ventilated with a respiratory device (Fabius, Draeger, Germany). The tidal volume was set at 10 mL/kg, the respiratory rate at 12–14 breaths/min, and the positive end-expiratory pressure was set at 5 mbar. The tidal volume and frequency were adjusted to keep end-expiratory CO_2_ within 35–40 mmHg and to maintain at least at 95% peripheral oxygen saturation.

**Figure 1:**
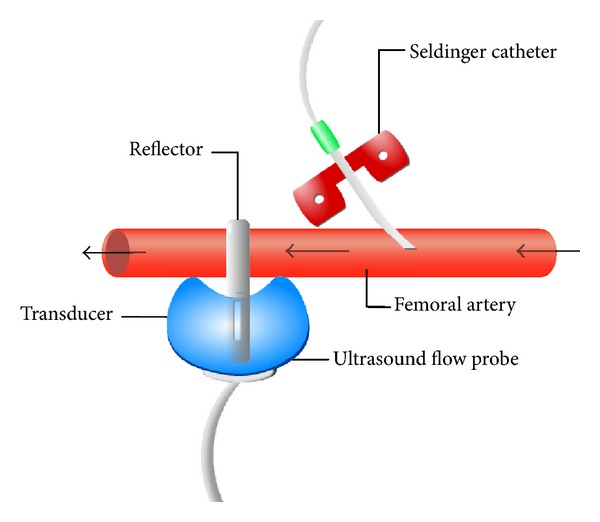
Positions of the ultrasound flow probe and catheter at the femoral artery. The arrows indicate the direction of blood flow.

A central venous catheter (3-Lumen-ZVK-Set, ARROWg^+^ard Blue, Arrow, Germany) was placed in the jugular vein on the right side of the neck of the pig. The central venous catheter was used for administration of contrast medium. Subsequently, the right or, if this was not possible, the left femoral artery was exposed. The femoral artery was catheterized proximally by the Seldinger technique to enable local administration of adenosine or CM. The metal-free, MRI-compatible catheter (Arterial Leader Cath, Vygon, France) was advanced proximally and fixed with several sutures. Distal to the catheter, an ultrasound flow probe (T206, Transonic Systems Inc., Ithaca, NY) was implanted around the femoral artery to measure blood flow (Figure 1). The probe was also fixed with sutures. The cable of the probe was led out of the wound in a direct way by tunneling of the subcutis. Gaps in the window of the transit time flow probe were carefully filled with ultrasound transmission gel and the wound was closed by sutures. In the first five pigs, correct implantation of the Seldinger catheter and the flow probe was tested by injection of adenosine over a period of approximately 30 min before transporting the pigs to the scanner room.

### 2.3. Protocol

First, the pig was transported into the scanner room. From the beginning of the transport the pig was ventilated with a portable medical ventilator (Oxylog, Dräger, Germany). The ventilator contained metal so it had to be placed outside the scanner room. The breathing tube therefore had to have length of 6 m. Due to the length of the tube, monitoring of end-expiratory CO_2_ with a monitoring device for capnography (Vamos, Dräger, Germany) was particularly important. The ventilator settings were adjusted for an end-expiratory CO_2_ level of 35–45 mmHg.

An MRI-compatible monitoring device (Veris, Medrad, Germany) was used for monitoring heart rate and oxygen saturation. The pig was placed in the supine position on the scanner table, which contains an integrated 32-channel spine coil (Siemens Magnetom Aera 1.5 T). A surface coil (Tim body coil, 18 RF channels) was placed on the hind limbs. The blood flow measurement data of the flow probe were continuously recorded using LabVIEW 2012, an A/D converter card (NI USB-6211), and a standard netbook with Windows XP.

First, morphological images were acquired without contrast medium administration for orientation (Figure 4). To avoid systematic errors the sequence of the different protocols was randomly changed. Thus, either baseline flow or increased flow following adenosine administration was performed first. A dynamic sequence with CM was used for measurements.

For vasodilatation, a perfusor was used for locally injecting adenosine via the Seldinger catheter in the femoral artery. The adenosine perfusion rate was individually and dynamically adapted to achieve similar blood flow levels in all pigs at each measurement time point. Steady blood flow occurred after approximately 5 min, and the first perfusion measurement was started. Thereafter, two further perfusion measurements with different perfusion levels and systemic CM administration were performed, followed by a perfusion measurement with local CM administration.

**Figure 2:**
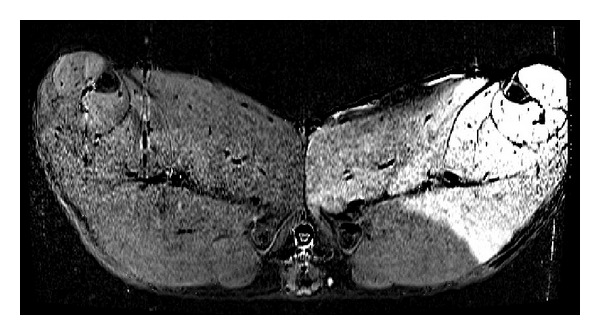
Difference image. T1-weighted images before and after local injection of contrast agent into the right femoral artery were acquired. There is significant brightening of the supply area.

**Figure 3:**
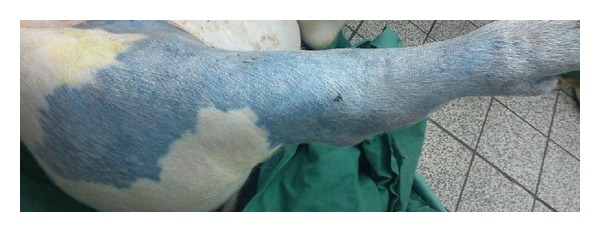
Photograph showing the supply area of the femoral artery after administration of Evans blue via the Seldinger catheter.

**Figure 4:**
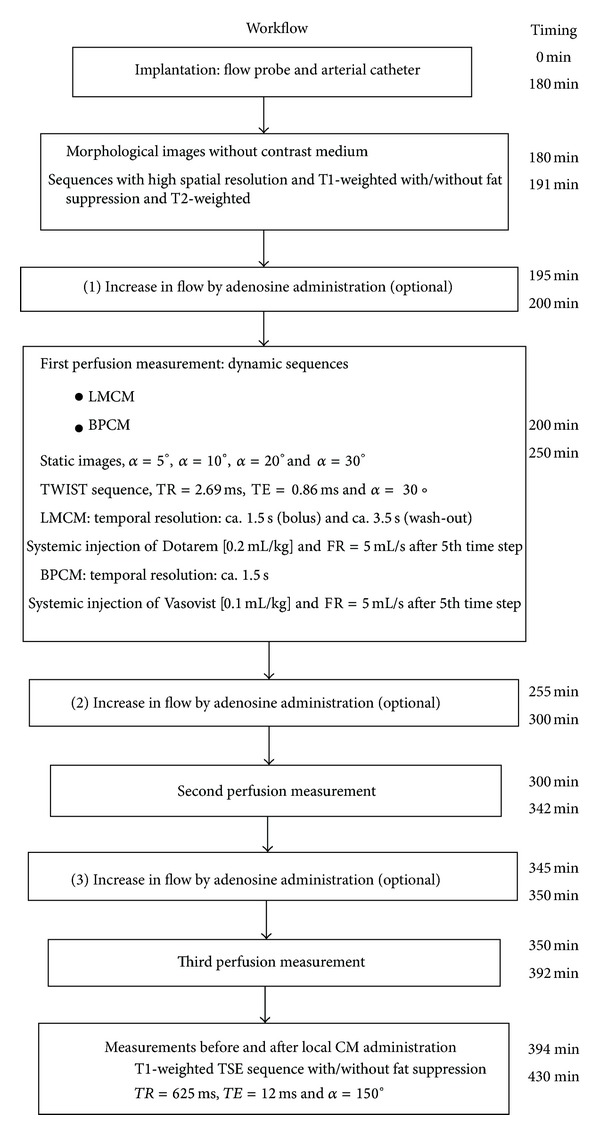
Representative example of workflow and timing in a single swine experiment.

Subsequently, a rapidly extravasating low-molecular weight contrast medium (LMCM) was injected locally to visualize the area supplied by the femoral artery (Figure 2). Finally, after completion of MRI scans, tissue was sampled from the supply area of the femoral artery for histological determination of blood volume and interstitial volume. These volumes were determined to serve as a reference for the parameters determined by MRI. Additionally, the supply area of the femoral artery was delineated by injection of Evans blue fluorescent dye (10 mL, *c* = 100 mg/mL) in the Seldinger catheter (Figure 3). The pig was euthanized under a higher level of anesthesia by the injection of T61 (0.3 mL/kg).

### 2.4. MRI Technique

First, unenhanced T1-weighted morphological images with and without fat suppression and T2-weighted images were acquired for orientation. The protocol included an axial T1-weighted turbo spin echo (TSE) sequence with fat suppression in the transverse plane with the following parameters: repetition time, TR = 625 ms, echo time, TE = 12 ms, flip angle, *α* = 150°, and voxel size, 0.9 × 0.9 × 7.0 mm^3^. Acquisition of these sequences was followed by perfusion measurements.

The protocol for perfusion measurement included a 3D gradient-echo sequence (Siemens syngo TWIST) with a high temporal resolution of approximately 1.5 s and the following parameters: TR = 2.69 ms, TE = 0.86 ms, *α* = 30°, voxel size of 2.89 × 4.5 × 2.9 mm^3^, 160 × 48 × 128 reconstruction matrix, frame, 100 and 250, frequency encoding in the axial direction, parallel imaging with GRAPPA with 32 central k-space lines and an acceleration factor of 6, and distribution of peripheral k-space lines to several images with keyhole technique.

The central 20% of the central k-space lines were scanned every time. For the first 100 measurements, the peripheral 80% of the k-space lines were split, and each line was sampled every fifth acquisition. After the fifth acquisition of the first dynamic sequence, 0.2 mL/kg body weight of LMCM (0.5 Mol/mL gadoteric acid, Dotarem, Guerbet, France) was injected via the central venous catheter, delivery rate: 5 mL/s. Then 20 mL of a 0.9% saline solution was injected with the same delivery rate. The subsequent 250 measurements are measured with a lower time resolution (approximately 6 s) without exploring the keyhole technique.

After acquisition of the pulse sequences described above, the next measurement was started using a blood pool contrast medium (BPCM) (0.25 mMol/mL gadofosveset trisodium, Vasovist Bayer Schering, Berlin, Germany/Ablavar Lantheus Medical Imaging, Inc., USA) that remains intravascular due to high protein binding. The BPCM was administered at a dose of 0.1 mL/kg body weight and an injection rate of 5 mL/s. CM administration was followed by injection of 20 mL 0.9% saline solution at the same rate. The dynamic acquisition was performed with identical parameters at 100 time points. Before each acquisition, static images were acquired using the TWIST sequence without the keyhole technique to create maps of baseline relaxivity and magnetization with flip angles of 5°, 10°, 20°, and 30°. After the first static acquisitions, adjustments were made and used for the subsequent static and dynamic acquisitions with the TWIST sequence. Time-dependent maps of relaxation rate changes were computed using the method of Li et al. [11].

Increased blood flow was induced by local injection of adenosine (Adenosin Life Medical, 5 mg/mL, Carinopharm, Germany) into the femoral artery using a syringe pump. The dose was chosen according to its flow-enhancing effect. After about 5 min, a steady blood flow, measured by the ultrasound flow probe, was achieved, and the measurement by MRI was started. After the fifth time step, the low-molecular weight CM was administered. Subsequently, a second measurement with the blood pool contrast medium (BPCM), which remains intravascular, was carried out. It was administered after the fifth time step as well.

A total of up to three perfusion measurements were performed per animal. After several measurement cycles, signal saturation due to contrast medium accumulation in tissues is expected to preclude reliable perfusion measurements. The order in which the different perfusion levels (baseline blood flow or increased blood flow induced by adenosine) were measured was varied in each pig. The first perfusion measurement was performed either at baseline blood flow or at blood flow enhanced by adenosine injection. The following measurements were carried out at either higher, lower, or identical blood flows compared with the first measurement. Over the entire series of experiments, the same number of different flow levels in each of the three measurements was achieved. This was done to compensate for systematic errors due to signal saturation resulting from contrast medium accumulation. After the perfusion measurements, the supply area of the femoral artery was determined by MRI. Anatomical images were used to generate difference images (Figure 2). First, anatomical images without CM were acquired. Then the scans with CM were done. A dose of 2 mL of the rapidly extravasating LMCM was administered locally at 0.1 mL/s. The protocol included an axial T1-weighted turbo spin echo sequence with fat suppression in the transverse plane with TR = 625 ms, TE = 12 ms, *α* = 150°, 0.877 × .877 × 8.4 mm^3^ voxel size, and 512 × 272 × 40 reconstruction matrix.

### 2.5. Patient Examination

To compare the data collected from the pig experiments with those in humans, arterial input function (AIF) relaxation rate change curves and tissue curves were generated from MRI data obtained in a 17-year-old female patient with a highly vascularized sarcoma in the left lower leg. MRI in this patient was performed on a 1.5 T system (Magnetom Symphony, Siemens, Erlangen, Germany). The DCE-MRI protocol included a 3D T1-weighted gradient-echo (GRE) sequence with TR = 3.64 ms, TE = 1.22 ms, *α* = 30°, 7 × 1.56 × 1.56 mm³ voxel size, and 4 × 144 × 192 reconstruction matrix. DCE-MRI using a BPCM and a LMCM was performed at 200 and 80 time points, respectively, with a time interval of 1.26 sec between images. Static MR images, acquired with flip angles of 5°, 10°, 20°, and 30°, with the other parameters identical to those of the dynamic sequences, were used to generate baseline magnetization and relaxation rate maps. These maps were used to calculate the dynamic change in relaxation rate from the dynamic scan using a flip angle method proposed by Li et al. [11].

## 3. Results

Eight pigs were used to establish the method of perfusion measurement by MRI with an ultrasound flow probe as a reference method and adenosine administration to enhance perfusion.

The flow measurements with the ultrasound flow probe were continuously recorded. During imaging with high flip angles, the signal measured by the probe was disturbed by the electromagnetic field. Therefore the flow probe measurements obtained before and after each MRI acquisition were averaged to estimate blood flow during MRI acquisitions.

**Figure 5:**
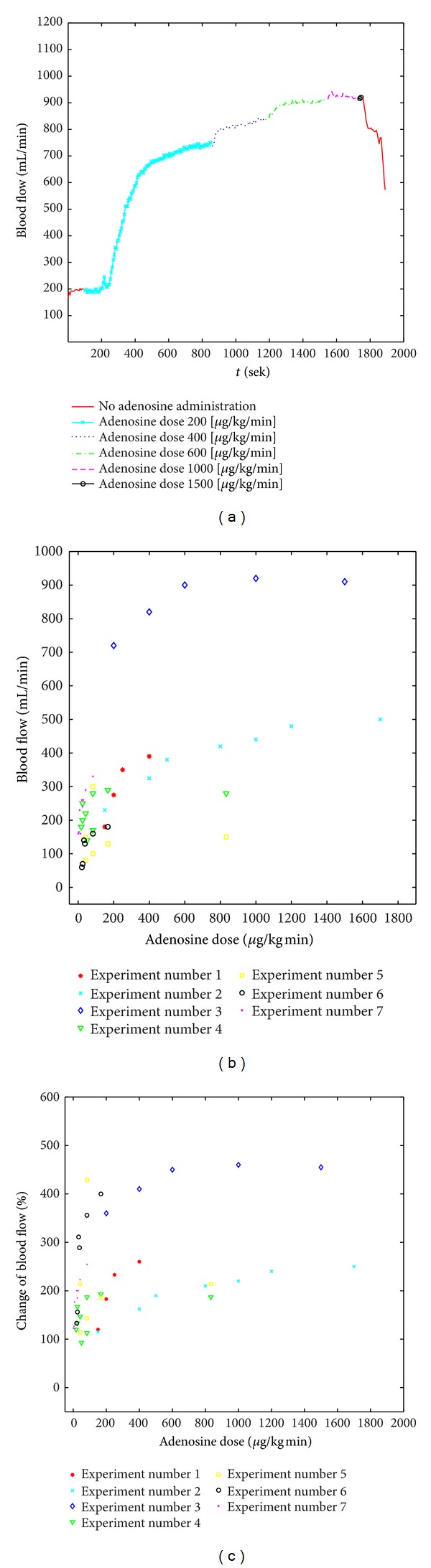
Increase in blood flow (mL/min) after local administration of adenosine in experiment number 5 (a). Distribution of adenosine doses (*μ*g/kg/min) with the corresponding blood flow levels (mL/min) in the first 7 experiments. The adenosine dose was set individually for each pig to achieve certain blood flows (b). Distribution of adenosine doses (*μ*g/kg/min) with the corresponding changes in blood flow [%] in the first 7 experiments. The increase in blood flow is given relativly to the baseline blood flow of each pig (c).

The desired levels of increased blood flow were accomplished by adjusting the adenosine dose based on its observed effect (Figure 5(a)).  Figure 5(b) shows the adenosine dosage (*μ*g/kg/min) with the corresponding blood flow (mL/min) for the first seven experiments. The adenosine dose was set individually for each pig to achieve certain blood flows.  Figure 5(c) shows the percentage change in blood flow in relation to the adenosine dose in seven pigs. All pigs had heart rates in the normal range of 60–80 beats/min. As soon as the increased blood flow remained constant over several minutes, the DCE-MRI measurement were performed. The supply area of the femoral artery was determined from difference images (Figure 2) generated by subtraction of anatomical images acquired before and after local CM administration. Precise knowledge of the area is essential, and, because of interindividual variation, it must be determined for each pig. Only the MRI perfusion values obtained in the supply area can be compared with the flow data measured by the flow probe in the femoral artery. Perfusion can be derived by using compartment-based pharmacokinetic models. Since the establishment of the method, evaluable data have been acquired in seven additional pigs.

**Figure 6:**
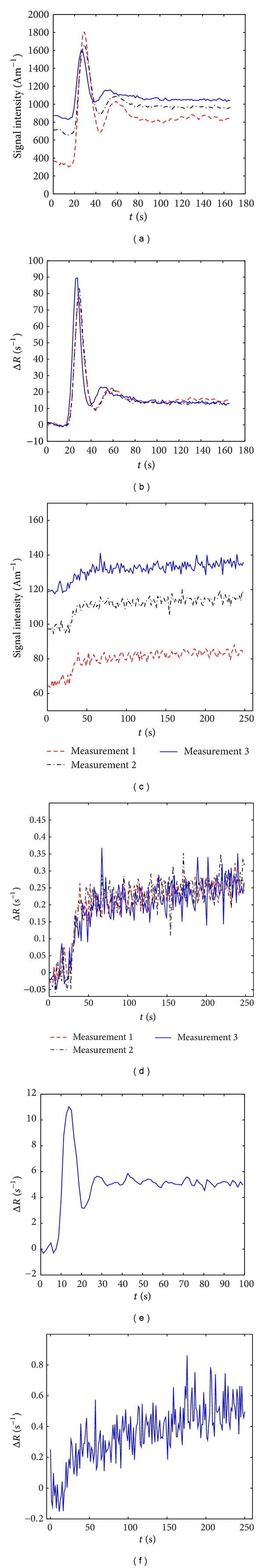
Signal curves of arterial input functions (AIF) of the aorta. The figure shows curves of three measurements after administration of the BPCM. The curves of the second and third measurements have a higher baseline signal than the first curve because of persisting effects of CM administered for the preceding measurement (a). Relaxation rate change versus time of the arterial blood measured in the aorta. The relaxation rate change curves were calculated with the method of Li et al. using the same data as for (a). The CM preload has no evident effect on the curves (b). Signal tissue curves of semitendinosus muscle using LMCM. As far as possible, no bones, skin, or great vessels were included (c). Corresponding to the method used in (c), relaxation rate change versus time curves were generated (d). Relaxation rate change versus time of the AIF of a 17-year-old female sarcoma patient measured in a vessel in the lower extremity using a BPCM (e). Relaxation rate change versus time of a tissue curve measured in a muscle of the lower extremity of the sarcoma patient using an LMCM (f).

Figure 6(a) presents signal intensity time curves of arterial input functions (AIF) of all three measurements in the aorta using the BPCM.  Figure 6(b) shows the corresponding maps of the time-dependent relaxation rate changes. The relaxation rate change curves were determined using the method of Li et al. [11]. The AIF could be well reproduced over three consecutive measurements despite a continuously increasing CM preload.

Figure 6(c) shows signal intensity tissue curves for the semitendinosus muscle after administration of LMCM. As far as possible, no bones, skin, and great vessels were included.  Figure 6(d) shows the corresponding relaxation rate change time curves. The signal curves of AIF and tissue show a higher baseline signal intensity in the second and third measurements than the first due to the effect of previously administered CM. The AIF signal intensity time curves of the second and third measurements show a flattened second pass and a stronger disturbance by noise.

The curves of the patient (Figures 6(e) and 6(f)) and the pig (Figures 6(b) and 6(d)) show a similar course. The AIFs of the pig (Figure 6(b)) were measured in the aorta. First and second passes are well recognizable. The AIF of the patient (Figure 6(e)) was measured in an artery of the lower extremity. After the second pass the curve remains on the same level. Overall, the patient curve shows a lower level. The AIF was measured in a smaller vessel, so that partial volume effects may have played a role. The tissue curves of patient and pig (Figures 6(d) and 6(f)) have the same order of magnitude.

Furthermore, the time intervals between first and second pass of the CM in the vessels were determined in five patients enrolled in a clinical study and in the eight pigs. The time between first and second pass corresponds to the circulation rate. The mean interval was 19.7 ± 4.8 s in the patients and 20.4 ± 5.1 s in the pigs, indicating that the data obtained in the pig experiments compare well with those in humans.

## 4. Discussion

The aim of our study was to develop an appropriate animal model to validate DCE-MRI-based perfusion measurements. The pig is a well-suited animal for experimental studies because its anatomy and physiology are similar to humans. This is confirmed by our findings. The pigs used in our experiments had heart rates of 60–80 beats/min, which corresponds to adult human heart rates. Blood volume of pigs is about 70–80 mL/kg and is also similar to that of humans (60–98 mL/kg) [12]. To provide steady blood flows for the perfusion measurements with or without adenosine it is essential to ensure stable blood circulation during measurements. Therefore, good anesthetic management is important. The relatively high susceptibility to stress the porcine cardiovascular system must be considered. The lower relative heart mass of swine compared with humans (approximately 0.3% versus 0.5–0.7%) suggests a lower stroke volume. Cardiac performance is considered to be lower [13]. The type and duration of anesthesia can affect blood circulation. During the experiments, 3 pigs developed bradycardia (about 45–55 beats/min) after 5–7 hours of anesthesia. This was probably due to the long duration of anesthesia and the cardiodepressive effects of propofol and fentanyl. Adenosine can also lower heart rates. However, the observed bradycardia was not temporally related to adenosine administration; therefore, the length of anesthesia appears to be the more likely cause. A low heart rate reduces peripheral perfusion. Therefore, it is important to keep the duration of anesthesia as low as possible. If bradycardia persists, it has to be corrected, for example, by administration of an anticholinergic drug such as atropine. The use of the femoral artery and its supply area for these experiments has several advantages over other vascular beds. The femoral artery is relatively easy to access by surgery. With a diameter of approximately 4.5–6 mm, the femoral artery is suitable for mounting a 6 mm flow probe. According to the manufacturer, at least 70% filling of the flow probe is desirable to obtain reliable measurements. Before choosing the femoral artery, we made an attempt to prepare the axillary artery of the right front leg. Due to the anatomical proximity of the axillary artery to the network of nerves of the brachial plexus, the axillary artery was less well accessible and it was not always possible to dissect the artery without damaging the nerves. Nerve preservation is important to ensure undisturbed innervation of blood vessels. Because of these problems, we finally opted for using the femoral artery in our experiments. Fixation of the ultrasound flow probe and the catheter with sutures to the musculature made the very fragile system more resilient during transport of the pigs to the scanner room.

Continuous local adenosine administration caused a sustained and steady increase in blood flow in the femoral artery that could be well controlled. The total adenosine doses administered were different among the pigs. This was deliberately accepted to achieve comparable perfusion values in the various phases of the experiments. However, with a constant supply of adenosine using a syringe pump, it was possible to establish steady blood flow after a few minutes. The respective blood flows were reproducible for each pig.

Achieving a steady perfusion level was essential because DCE-MRI acquisitions at high flip angles interfered with blood flow measurement using the ultrasound flow probe.

To overcome this limitation, blood flow during MRI acquisition was calculated by averaging the probe-based flows measured before and after MRI acquisition. Our results show that the pig model used in the present study is well suited for DCE-MRI-based perfusion measurement.
